# Spinal postural variability relates to biopsychosocial variables in patients with cervicogenic headache

**DOI:** 10.1038/s41598-021-93138-3

**Published:** 2021-07-02

**Authors:** Sarah Mingels, Wim Dankaerts, Ludo van Etten, Liesbeth Bruckers, Marita Granitzer

**Affiliations:** 1 REVAL Rehabilitation Research Centre, Biomedical Research Institute, Faculty of Rehabilitation Sciences, Hasselt University, 3500 Hasselt, Belgium; 2 Musculoskeletal Research Unit, Department of Rehabilitation Sciences, Faculty of Kinesiology and Rehabilitation Sciences, Leuven University, 3000 Leuven, Belgium; 3 Department of Biometrics, Zuyd Hogeschool, 6419 Heerlen, The Netherlands; 4 Interuniversity Institute for Biostatistics and Statistical Bioinformatics, Hasselt University, 3500 Hasselt, Belgium

**Keywords:** Headache, Pain

## Abstract

Patients with cervicogenic headache (CeH) showed lower spinal postural variability (SPV). In a next step, the complex character of such SPV needs to be analysed. Therefore, variables influencing SPV need to be explored. A non-randomized repeated-measure design was applied to analyse relations between biopsychosocial variables and SPV within a CeH-group (n = 18), 29–51 years, and matched control-group (n = 18), 26–52 years. Spinal postural variability, expressed by standard deviations, was deducted from 3D-Vicon motion analysis of habitual spinal postures (degrees). Interactions between SPV and pain processing, lifestyle, psychosocial characteristics were analysed. Pain processing characteristics included symptoms of central sensitization (Central Sensitization Inventory), (extra)-cephalic pressure pain thresholds (kPa/cm^2^/s). Lifestyle characteristics included sleep quality (Pittsburgh Sleep Quality Index), physical activity, screen-time, sedentary-time (hours a week), position (cm) and inclination (degrees) of the laptop (= desk-setup). Psychosocial characteristics included degree of depression, anxiety and stress (Depression Anxiety Stress Scale-21), impact of headache on quality of life (Headache Impact Test-6). Spinal postural variability related significantly to intrinsic (stress, anxiety, extra-cephalic pressure pain thresholds, sleep-duration) and extrinsic (desk-setup, screen-time) variables in the CeH-group. In the control-group, SPV related significantly to extra-cephalic pressure pain thresholds. Spinal postural variability related to diverse variables in the CeH-group compared to the control-group. More research is needed into a possible causal relationship and its clinical implication.

## Introduction

Motor variability is hypothesized to fulfil a beneficial role in preventing the development of overuse injuries and pain^1^. Its dynamic spectrum ranges from subtle motor adaptations, to complete avoidance of movement as response to (impending) pain^2–4^. However, the relationship between pain and motor variability is complex. Too little or too much variability could harm tissue^2^. Secondly, motor responses to pain can be highly variable between, and probably within individuals due to the unique character of motor variability^3,5,6^.

Although motor variability can be individual- and task-specific driven, generally accepted theories predict rather stereotypical motor responses to pain^7,8^. The *pain adaptation theory* supports decreased activity of muscles producing painful movements, and facilitation of antagonists^7^. The *pain-spasm-pain cycle* or *vicious cycle theory* on the other hand, predicts increased muscular activity with at the end more pain induced by the process of muscle ischaemia^8^. A more *contemporary theory* of motor responses to pain postulates that pain may force a patient to adopt a protective and less variable posture^3,4^. This contemporary theory suggests that pain aims to protect the painful or threatened part of the body by constraining movement. Falla et al. demonstrated that such protective motor adaptations even occur at a distance from the site of pain, with participants with neck pain walking with a stiffer (i.e. decreased rotation) trunk^3,9^. It is hypothesized that the overall goal of this protective behaviour is to enhance stability by resisting intervertebral perturbations^3,10^. Understanding motor variability in the context of pain could certainly guide management for recovery and restoration of function^2^. In this regard, motor variability is already studied in patients suffering from work-related musculoskeletal overuse injuries, low back pain, and recently cervicogenic headache (CeH)^1,4–6,8,11^. Recently lower motor variability during laptop-work, referred to as spinal postural variability (SPV), was demonstrated in patients with CeH^11^.

Focusing on postural research in CeH might become of special interest within the current context of COVID-19^12^. Prevalence of CeH might increase as a consequence of COVID-19. Two separate pathways could support such hypothesis: (1) indirectly, due to the forced ‘working from home’ policy resulting in a less optimal ergonomic work-setting, and (2) directly, due to being more sedentary in being sick and/or recovering from the COVID-19 infection. Both pathways might relate to less optimal and less variable postural behaviour, potentially contributing to developing CeH^13–15^.

As a consequence, it might be hypothesized that decreased cervical SPV, due to stereotypical motor behaviour and maintaining a posture^1,16^, might contribute to CeH.

Though research has been performed to analyse SPV in patients with CeH, research into biopsychosocial (BPS) variables such as pain, psychosocial, and lifestyle characteristics, influencing such variability is not available for this population^11^. Identifying such variables could attribute to a better understanding of the complex multi-dimensional character of SPV, its relation to CeH and how to prevent/manage it in the future.

Therefore, the main objective of the current study was to explore if BPS variables such as pain, psychosocial, and lifestyle characteristics significantly relate to SPV in patients with CeH compared with healthy controls.

## Methodology

### Design

Non-randomized analysis of relations between BPS variables and SPV during the 30-min-laptop-task within the CeH-group and matched control-group.

### Sample size

An a priori sample size was estimated (G*Power 3.1.9.4, Kiel, Germany) based on repeated-measures at five time-points (F-test, between-factors) of the forward head posture (FHP) (mean degrees and standard deviation) during a laptop-task. A total of 30 participants (15 participants per group, power 80%; α = 0.05) was required to detect a mean difference of 3.5° (± 1.3) in FHP between the headache-group and control-group^17^.

### Participants and ethics

Participants for both the CeH-group and the control-group were recruited between January 2018 and August 2019. The neurological staff at the headache departments of the AZ Vesalius hospitals (Tongeren and Bilzen, Belgium) identified and referred participants meeting the study’s inclusion criteria for CeH (see below for details). Additionally, a general call was launched at the Hasselt University, Zuyd Hogeschool (The Netherlands) and physiotherapy practices. Each potential participant for the CeH-group had to be declared eligible by a neurologist (external member of the research team). The neurologist was involved in determining the inclusion and exclusion criteria, confirmed the diagnosis of CeH based on the International Classification of Headache Disorders-3 (ICHD-3) criteria^18^, and referred eligible participants with CeH to the principal researcher (= physiotherapist, degree in manual therapy, > 10 years of experience).

Potential participants for the control-group were recruited by convenience sampling, word-of-mouth advertising within the Zuyd Hogeschool, and in the personal network of the involved researcher (Appendix A, Figure A.1).

*Inclusion criteria for the CeH-group* were: Dutch-speaking participants between 18 and 55 years, body mass index (BMI) between 18.5 and 24.9 kg/m^2^, diagnosed with secondary episodic CeH according to the ICHD-3^18^ by a neurologist, normal cognitive capacity (Mini Mental State Examination test score of 30), habitual laptop-use (minimum of 7 h/week) (Appendix A, Table A.1). *Inclusion criteria for the control-group were*: Dutch-speaking healthy participants (i.e. no known painful conditions or serious pathologies) between 18 and 55 years, BMI between 18.5 and 24.9 kg/m^2^, normal cognitive capacity (Mini Mental State Examination test score of 30), habitual laptop-use (minimum of 7 h/week) (Appendix A, Table A.1). *Exclusion criteria for both groups* were: pregnancy, physiotherapy for head- or neck-related disorders in the past month before the start of the study, serious pathology (musculoskeletal, neurological, endocrine, cardiovascular, psychiatric), medication overuse (intake of NSAID’s, opioids, acetylsalicylic acid, triptans, simple analgesics for > 10 days/month > 3 months), smoking, history of neck/head trauma, orthodontics (Appendix A, Table A.1).

Nineteen participants were recruited and selected to compose the CeH-group (Appendix A, Figure A.1). These participants were given a four-week headache-diary which had to be completed before the start of the study. The control-group was matched for age, gender, ethnicity and socio-economic status (level of education, job).

The current study was part of phase 1 of a larger project (hence the larger sample size) which was registered as an observational study at ClinicalTrials.gov (registered on 02/09/2016, NCT02887638). The Medisch Ethische Toetsings Commissie of Zuyderland and Zuyd Hogeschool (NL. 55720.09615) and the Comité Medische Ethiek of the Ziekenhuis Oost-Limburg (B371201423025) granted approval to execute the experimental protocol. Eligible participants had to read and sign the informed consent before officially being enrolled. Protection of personal data was legally determined by the Belgian law of December 8th 1992. All test procedures involving human participants were in accordance with the ethical standards of the institutional research committees and with the 1964 Helsinki Declaration and its later amendments. An informed consent was obtained from the participant in Fig. 4 for publication of the identifying image. The current study was prepared according to the Strengthening the Reporting of Observational Studies in Epidemiology (STROBE) guidelines.

### Measurements, outcomes and instruments

#### Primary outcome: spinal postural variability (SPV)

*Spinal postural variability* expresses variation in habitual spinal posture (= the position of the spine expressed in degrees) of the upper- and lower-cervical (UCx, LCx), thoracic (UTx, LTx), and lumbar (ULx, LLx) spine. Habitual spinal postures were evaluated with a 3D-Vicon motion analysis system (Vicon Motion Systems Ltd., Oxford, UK) and Vicon Nexus software (version 2.1.1, Oxford Metrics Ltd., Oxford, UK)^19^ for recording, data acquisition, storage, and gap filling during the 30-min-laptop-task^11,20,21^. The accuracy of the system is < 1° and < 1.5° root mean square in static and dynamic angular measurements, respectively^20–22^. *Spinal postural variability* of the UCx, LCx, UTx, LTx, ULx, and LLx, expressed by the standard deviation (SD), was deducted from the habitual spinal postures during the 30-min-laptop-task.

#### Secondary outcomes: biopsychosocial (BPS) variables

Interpretations of the outcomes are presented in Appendix B (Tables B.1 to B.5).

##### Pain processing characteristics

1. Pressure Pain Thresholds (PPTs) (kPa/cm^2^/s) of the bilateral suboccipitals (cephalic), erector spine at L1 (extra-cephalic), and tibialis anterior (extra-cephalic) were measured with an electronic pressure algometer (Somedic AB, Stockholm, Sweden)^23–27^. Pressure Pain Threshold is defined as the minimal amount of pressure that elicits pain. Hypersensitivity over remote, extra-cephalic sites is considered to be a sign of central sensitization. Intra-rater reliability of cervical PPT-measurements are moderate to good (ICC 0.79–0.90) in healthy participants, and good to excellent (ICC 0.82–0.99) in patients with headache^24,26,28^. Intra-rater reliability of erector spine PPT-measurements are excellent (ICC 0.95–0.98) in healthy participants^29^. Intra-rater reliability of tibialis anterior PPT-measurements are excellent in healthy participants (ICC 0.94), and patients with neck pain (ICC 0.97)^24^.
2. Symptoms of central sensitization were questioned via the Dutch Central Sensitization Inventory (CSI)^30^. Test–retest reliability (ICC 0.82–0.97), and internal constancy (Cronbach’s α 0.87–0.91) are good to excellent^31,32^.
3. Headache characteristics, i.e. headache-intensity (mean 100 mm Visual Analogue Scale (VAS) per attack during a month), duration (mean hours per attack during a month), and frequency (days per month) were extracted from the headache diary (Belgian Headache Society)^33^. Additional information on referred pain from the neck (yes/no) was obtained through the anamnesis.

The Numeric Pain Rating Scale (NPRS) was used to capture headache-intensity pre and post the 30-min-laptop-task^34–37^.

##### Lifestyle characteristics

1. Sleep quality was assessed via the Dutch Pittsburgh Sleep Quality Index (PSQI) which is a standardized, valid and reliable self-reported 1-month recall questionnaire^38–41^. The index differentiates poor from good sleepers by measuring seven components: subjective sleep quality, sleep latency, sleep duration, habitual sleep efficiency, sleep disturbances, use of sleeping medication, and daytime dysfunction.
2. Physical activity (mean hours a week), *screen-time* (mean hours a week of computer-use), and sedentary-time at home and during work (mean hours a week) were extracted from a customized one-week recall questionnaire.

##### Psychosocial characteristics

1. Degree of depression, anxiety and/or stress was estimated by the Dutch Depression Anxiety Stress Scale-21 (DASS-21), a self-reported one-week recall questionnaire. Internal constancy for the three subscales is good to excellent (Cronbach’s α 0.91, 0.84, and 0.90, respectively)^42–44^. Each of the three sub-scales contain seven items. The depression subscale assesses dysphoria, hopelessness, devaluation of life, self-deprecation, lack of interest, anhedonia and inertia. The anxiety subscale estimates autonomic arousal, skeletal muscle effects, situational anxiety, and subjective experience of anxious affect. The stress subscale evaluates difficulty in relaxing, nervous arousal, and being easily upset and impatience.
2. Impact of headache on quality of life was assessed with the Dutch Headache Impact Test-6 (HIT-6)^45–48^. Internal consistency is good to excellent (Cronbach’s α 0.89–0.90)^46^. The HIT-6 evaluates the impact of headache on daily activities: ability to function at work, school, home, and in social situations.

### Procedure

A condition to be measured was a score of < 3 on the 11-point NPRS for headache-intensity on the test day^3^. Participants were asked not to take analgesics, muscle relaxants, and caffeine-containing beverages 24 h prior to the measurements. Prophylactic treatment(s) remained unchanged. Measurements were performed in a real-life set-up with a constant room temperature of 25°C at the motion laboratory of Zuyd Hogeschool (Heerlen, The Netherlands), and executed by the principal researcher.

**Figure 1:**
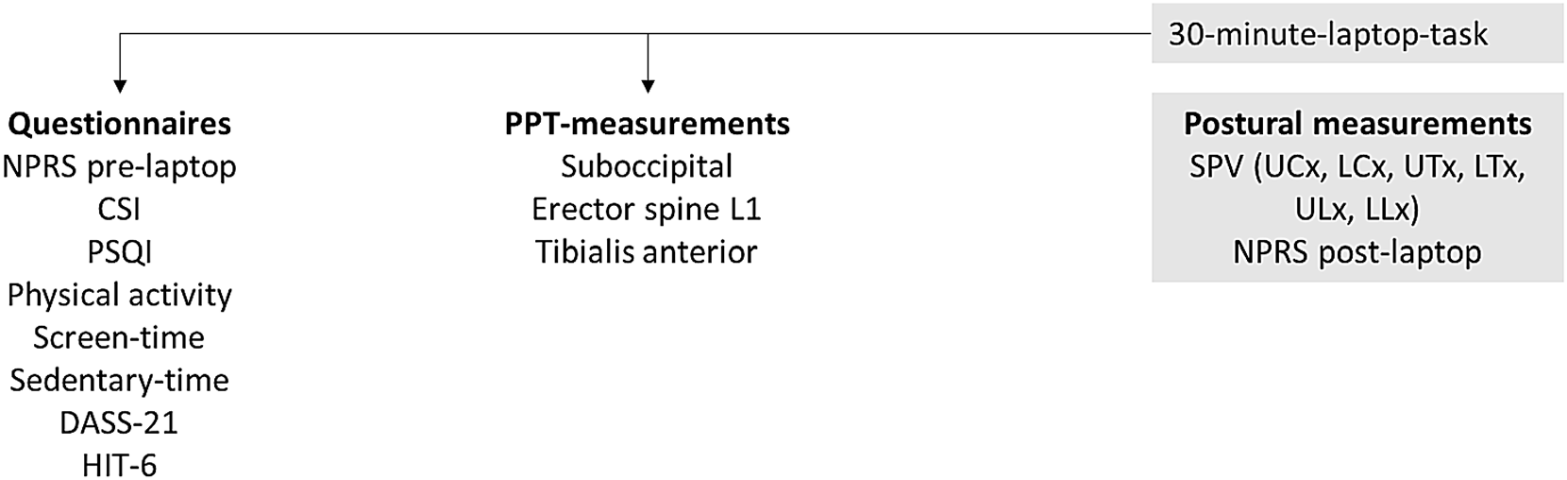
Chronology of the test procedure for both groups. We refer to our previous work and Appendix C concerning the SPV-measurements (grey box)^11^ (NPRS = Numeric Pain Rating Scale, CSI = Central Sensitization Inventory; PSQI = Pittsburgh Sleep Quality Index; DASS = Depression, Anxiety, Stress Scale; HIT = Headache Impact Test; PPT = Pressure Pain Threshold; SPV = Spinal Postural Variability) (Microsoft PowerPoint 2016, version 1, Microsoft Corporation).

Questionnaires were completed first, followed by PPT-measurements, and final habitual spinal posture measurements (Fig. 1). Headache-intensity and neck pain intensity (NPRS) were questioned pre and post the 30-min-laptop-task in both groups.

A standardized protocol was used to measure PPTs of the bilateral suboccipitals, erector spine at L1 (neutral prone position) and tibialis anterior (seated with 80° knee-flexion)^24,25^. Pressure was perpendicular applied directly on the muscle belly, staring at 0 to maximal 1000 kPa, using a 1 cm^2^ probe with a slope of 30 kPa/s^49,50^. Participants were instructed to push the stop-button when the sensation of pressure changed into pain. An exercise trial was once performed on the right thigh before actually measuring. Measurements were executed twice (ICC 0.86–0.99) after a 5-min interval in a standardised column-wise order: suboccipital left, erector spine at L1 left, tibialis anterior left, suboccipital right, erector spine at L1 right, tibialis anterior right^26,50–52^. Mean values of two measurements on each location were used for further statistical analysis^24,53^.

**Figure 2:**
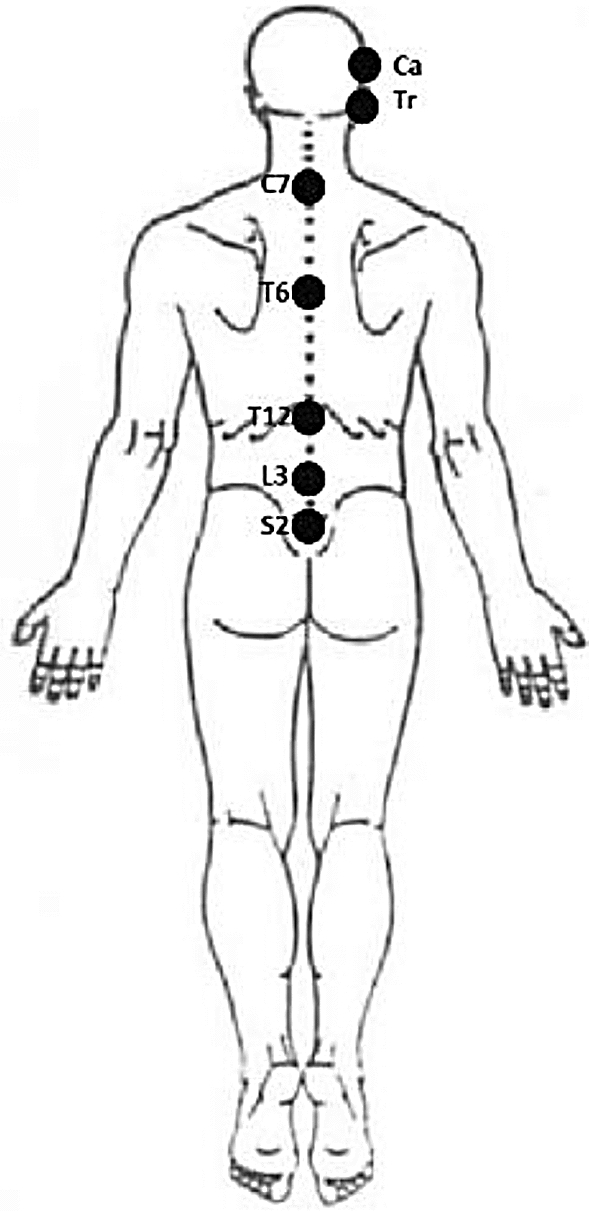
Visualisation of the marker-placement to model the spine (Tr = Tragus; Ca = Canthus).

Spinal postural variability of the UCx, LCx, UTx, LTx, ULx, and LLx during the 30-min-laptop-task were measured according to procedures described in our previous work^11^ (Appendix C). Figure 2 provides a visualization of the marker-placement used to determine UCx, LCx, UTx, LTx, ULx, and LLx angles. Spinal postural variability was deducted from the angular measurements.

### Statistics

Analysis was done via JMP Pro 14 and SAS 9.4. Two-tailed tests at 5% level of significance were reported.

#### Demographics and group characteristics

Unpaired t-tests were used to compare continuous variables. Contingency tables (Fisher’s exact test) were composed to compare distributions of categorical variables (proportions) between groups.

*Spinal postural variability* was deducted from linear mixed models for repeated measures, based on the lowest root-mean-square error, with dependent variables (habitual spinal posture), fixed (time, baseline, group), random (individual, time) effects, and an autoregressive covariance structure [AR(1)]: Y_ij_ = β_0_ + β_1_X_1ij_ + ⋯ + β_k_X_kij_ + μ_i_ + ε_ij_ (Appendix D, Table D.1).

*Spinal postural variability* was deducted from the random-intercept of this mixed model. To express SPV, mean SDs were calculated per habitual spinal posture (UCx, LCx, UTx, LTx, ULx, LLx) per group at t0 (since, based on the mixed model, time was not related to SPV) (Appendix D, Table D.2). Effect sizes (ES) to quantify differences in SPV between groups (Cohen’s d) were reported and interpreted as: ≤ 0.20 small, 0.21–0.49 moderate, 0.50–0.79 medium, ≥ 0.80 large ES^54^.

Next, multiple regression models with a stepwise approach were built per group to identify possible *BPS dependent variables* related to the SDs of the UCx, LCx, UTx, LTx, ULx, and LLx. Dependent variables were ranked from most to least influence based on R^2^ and standardized coefficients in case of model significance. Conditions to apply linear models (i.e. normal distribution, linearity, homoscedasticity of residuals, no multicollinearity (Variance Inflation Factor (VIF) < 4.00) between variables) had to be met.

*Relations between the independent variables* age, BMI, the interaction between age and BMI (age*BMI), headache-intensity (continuous), socioeconomic status (categorical), and SDs of the UCx, LCx, UTx, LTx, ULx, LLx (dependent continuous) were evaluated via multiple linear regression: Y_i_ = β_0_ + β_1_X_i1_ + β_2_X_i2_ + β_3_X_i1_X_i2_ + ⋯ + β_k_X_ik_ + ε_i_ (Appendix E, Tables E.1 and E.2). Conditions to apply linear models had to be met.

*Mean (SD) headache-intensity (NPRS)* was compared pre and post the laptop-task by using the paired t-test. Effect sizes to quantify differences in change of headache-intensity from pre- to post-laptop-task were reported (interpretation see above)^54^.

## Results

### Demographics and group characteristics (Table 1)

**Table 1:** Demographics and group characteristics of the CeH-group (n = 18) and control-group (n = 18).

	CeH-group	Control-group	*p*
Age (y), mean (SD) [CI]	40.2 (10.9) [34.6; 45.8]	39.2 (13.1) [32.7; 45.7]	0.80^†^
BMI (kg/m^2^), mean (SD) [CI]	23.5 (3.2) [21.9; 25.1]	23.2 (3.2) [21.6; 24.8]	0.76^†^
**Marital status, n (%)**			1^‡^
Married	9 (50)	9 (50)	
Living together	5 (27.8)	4 (22.2)	
In a relation (not living together)	2 (11.1)	3 (16.7)	
Single	2 (11.1)	2 (11.1)	
**Socioeconomic status, n (%)**			
*Employment*			0.65^‡^
Student	2 (11.1)	3 (16.7)	
Working	16 (88.9)	15 (83.3)	
Services	14 (87.5)	13 (72.2)	
Self-employed	2 (12.5)	2 (12.5)	
*Level of education*			1^‡^
Secondary studies	2 (11.1)	2 (11.1)	
Graduate school or university	16 (88.9)	16 (88.9)	
**Dominant hand, n (%)**			0.22^‡^
Left	3 (16.7)	0	
Right	15 (83.3)	18 (100)	
Screen-inclination laptop (°), mean (SD) [CI]	115.3 (5.5) [112.4; 118.1]	112.4 (11.3) [106.8; 118.1]	0.35^†^
Distance laptop—table edge (cm), mean (SD) [CI]	10.1 (5.5) [7.4; 12.8]	9.9 (4.5) [7.7; 12.1]	0.89^†^

SD = Standard Deviation; CI = 95% Confidence Interval; n = number participants; y = years; ^†^ = unpaired *t *test; ^‡^ = contingency table for categorical variables (Fisher’s exact test); cm = centimetre.

Demographics and group characteristics were comparable between the groups. Two participants (one in the CeH-group, one in the control-group) had to be excluded because of technical artefacts during the postural measurements. Age, BMI, the interaction between age and BMI (age*BMI), level of education, employment and headache-intensity did not significantly relate to SPV (Appendix E, Tables E.1 and E.2).

### Headache characteristics (Table 2)

**Table 2:** Headache characteristics of participants with CeH (n = 18) and healthy controls (n = 18).

Headache characteristics	CeH-group			Control-group	
Headache duration, mean hours/episode (SD) [CI]	4.1 (1.6) [3.3; 4.9]			N/A	
General headache intensity, mean VAS/episode (SD) [CI]	61 (14) [54.4; 67.4]			N/A	
Headache-frequency, median days/month [IQR]	11 [10; 15.8]			N/A	
**Referred pain from the neck, n (%)**					
Yes	18 (100)			0	
No	0			18 (100)	

	Pre	Post	p* (ES)	Pre	Post
**Instantaneous headache-intensity**					
Mean NPRS (SD)	0.7 (1)	3.6 (2.1)	< 0.001 (0.89)	0	0
[CI]	[0.3; 1.2]	[2.6; 4.7]			
**Instantaneous neck-pain-intensity**					
Mean NPRS (SD)	0	3.6 (2.1)	< 0.0001 (1.71)	0	0
[CI]	0	[2.6; 4.7]			

n = number participants; SD = Standard deviation; CI = 95% Confidence interval; VAS = 100 mm Visual analogue Scale; NPRS = 11-point Numeric Pain Rating Scale; IQR = 25–75% Interquartile range; N/A = Not applicable; ES = Effect size; Pre = pre laptop-task; Post = post laptop-task; * = paired *t *test; Bold numbers = *p* < 0.05.

Participants with CeH suffered from an episodic, moderate to severe intense headache with a mean duration of 4.1 h/episode. Referred pain from the neck was reported by all 18 participants. No headache or neck pain were reported at the start of the test procedure.

Mean headache-intensity (SD) was significantly (*p* < 0.001, ES 0.89) higher at the post laptop-task measurement [NPRS 3.6 (2.1)] compared to at the measurement before the laptop-task [NPRS 0.7 (1)]. This difference is larger than the minimal clinical meaningful difference of 2.5^37^. Participants in the control-group did not report headache or neck pain pre and post-laptop task.

### Spinal postural variability

#### Evolution of SPV during the 30-min-laptop-task (Fig. 3)

**Figure 3:**
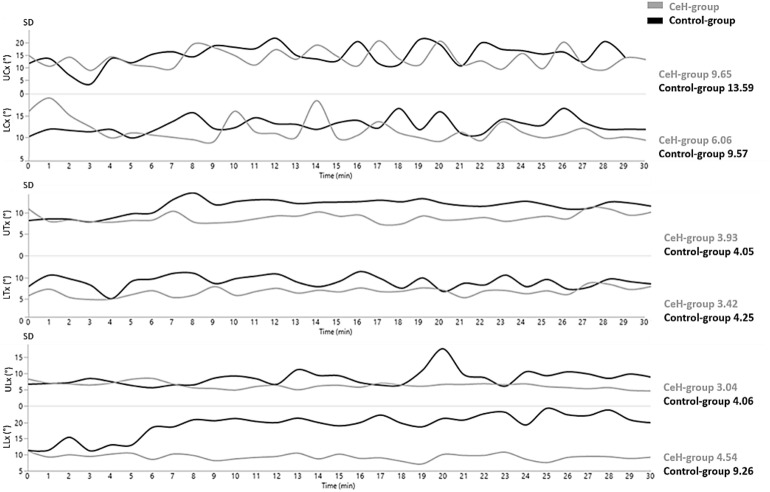
Visualisation of SPV (expressed by SDs) during the 30-min-laptop-task in the CeH-group and control-group (SD = Standard Deviation; ° = degrees; min = minute; mean SDs are reported on the right chart side).

Figure 3 visualises fluctuations of SDs of the UCx, LCx, UTx, LTx, ULx, and LLx per minute during the 30-min-laptop-task in the CeH-group (grey) and control-group (black). The evolution of SDs was rather linear (time-effect *p* > 0.05, Appendix D, Table D.2), meaning that time did not relate to SPV. Mean SDs were generally lower in the CeH-group compared to the control-group^11^.

### Comparison of SPV between the CeH-group and control-group (Table 3)

**Table 3:** Comparison of SPV in the CeH-group (n = 18) and control-group (n = 18).

SPV (SD) [CI]	CeH-group	Control-group	*p*^†^ (ES)
UCx	9.65 (4.2) [1.07; 18.23]	13.59 (4.1) [5.27; 21.92]	0.50
LCx	6.06 (3.68) [4.17; 7.96]	9.57 (10.21) [4.32; 14.82]	0.20
p*	0.35	0.36	
UTx	3.93 (1.83) [2.99; 4.87]	4.05 (2.8) [2.61; 5.48]	0.88
LTx	3.42 (2.18) [2.25; 4.58]	4.25 (3.17) [2.62; 5.89]	0.38
p*	0.45	0.85	
ULx	3.04 (1.46) [2.29; 3.79]	4.06 (3.4) [2.31; 5.8]	0.28
LLx	4.54 (2.96) [3.02; 6.07]	9.26 (6.89) [5.72; 12.81]	< 0.001 (0.89)
p* (ES)	0.034 (0.39)^1^	< 0.001 (0.96)	

SD = Standard deviation; CI = 95% Confidence interval; ^†^ = unpaired t-test; * = paired *t *test; ES = Effect size; ^1^ = 1-sided *t *test *p* = 0.017; Bold numbers = *p* < 0.05.

Spinal postural variability of the LLx was significantly lower (*p* < 0.001) in the CeH-group compared to the control-group, which indicated that these participants moved less during the 30-min-laptop-task. Spinal postural variability of the other spinal postures was, however not significant, consistently lower in the CeH-group compared to the control-group. Spinal postural variability of the ULx was significantly lower compared to LLx in both the CeH-group and control-group (*p* = 0.034, *p* < 0.001, respectively), indicating that participants moved less at the ULx compared to the LLx.

#### Relation between BPS variables and SPV

Detailed results on pain processing, psychosocial and lifestyle characteristics are presented in Appendix F (Tables F.1 and F.2).

**Figure 4:**
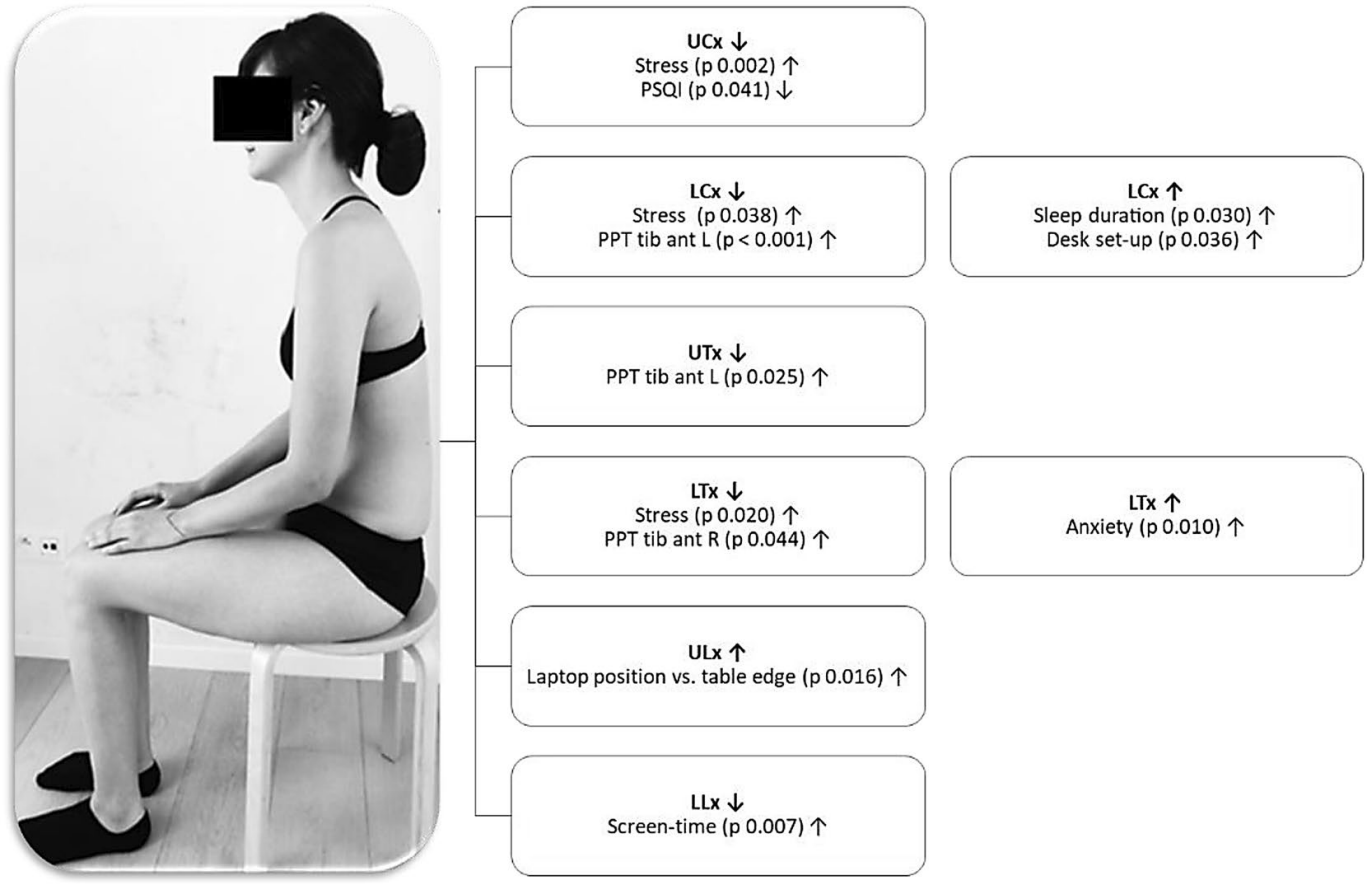
Summary of significant relations between BPS variables and SPV in the CeH-group (tib ant = tibialis anterior; L = Left; R = Right; PSQI = Pittsburgh Sleep Quality index; Arrows ↓ and ↑ refer to the direction of the relation, namely decrease (less movement) and increase (more movement), respectively).

*Biopsychosocial variables that were significantly related to SPV in the CeH-group *are summarized below (x represents the dependent variable in the equation). Interpretation of these results is visualized in Fig. 4:

- Spinal postural variability of UCx decreased significantly if the level of stress increased (14.5–9.71x, *p* = 0.002), and sleep quality decreased (12.64–7.99x, *p* = 0.041).
- Spinal postural variability of LCx decreased significantly with an increase in stress level (6.18–1.97x, *p* = 0.038), and in PPTs of the tibialis anterior left (14.46–0.02x, *p* < 0.001), SPV of LCx increased significantly with increasing sleep duration (− 7.69 + 1.99x, *p* = 0.030), and if the laptop position was further referred to the table-edge (x_1_) with a larger inclination of the laptop-screen (x_2_) (− 30.59 + 0.42x_1_ + 0.28x_2_, VIF 1.19, *p* = 0.036).
- Spinal postural variability of UTx decreased significantly with increasing PPTs of the tibialis anterior left (6.79 – 0.008x, *p* = 0.025).
- Spinal postural variability of LTx decreased significantly with increasing PPTs of the tibialis anterior right (6.25 – 0.007x, *p* = 0.044), and levels of stress (5.69 – 2.59x, *p* = 0.020), SPV of LTx increased significantly with an increase in level of anxiety (3.35 + 2.34x, *p* = 0.010).
- Spinal postural variability of ULx increased significantly if the laptop was further positioned referred to the table edge (1.48 + 0.15x, *p* = 0.016).
- Spinal postural variability of LLx decreased significantly with increasing screen-time (6.68 – 2.8x, *p* = 0.007).

*Biopsychosocial variables that were significantly related to SPV in the control-group *are summarized below (x represents the dependent variable in the equation):

- Spinal postural variability of LCx increased significantly with an increase in PPTs of the tibialis anterior right (− 10.56 + 0.05x, *p* < 0.001), and PPTs of the suboccipitals right (0.4 + 0.03x, p 0.002). Further, SPV of LLx increased significantly with increasing PPTs of the tibialis anterior right (− 3.29 + 0.03x, *p* = 0.002).

## Discussion

The main objective of the current study was to explore SPV in patients with CeH from a BPS point of view. Although SPV of these patients is lower compared to healthy controls^11^, research into multi-dimensional variables related to such SPV is non-existent. Since motor variability results from continuous motor adaptations as response to individual demands of a task, pain or other multi-dimensional factors, a BPS approach seems essential to unravel the potential relationships with SPV in patients with this musculoskeletal pain condition^55,56^.

### Lower spinal postural variability in patients with CeH

Motor variability (including among others SPV) was neglected for a long time by the scientific community. It was even referred to as ‘noise’ or ‘interference’^57^. More recently the role of motor variability retrieved attention in natural processes such as movement adaptation^58,59^. The latter should enable an individual to respond adequately to functional tasks and environmental constraints^60^, and prevent musculoskeletal overuse injuries and pain^1,57,61^. In general, it is presumed that higher motor variability is a healthier state, and lower motor variability relates to musculoskeletal disorders (MSDs). It should however be kept in mind that ‘too much’ motor variability can result in overuse-injuries^62^.

A renewed *redundancy theory* concerning motor variability was introduced based on the principle of abundance^63^. This theory postulates that there is no single optimal solution to resolve a task, but rather many similar solutions. This is referred to as ‘goal equivalent variability’^59^. Motor variability at both articular and muscular level, is an inherent characteristic of movement and is hypothesized to serve a protective role against developing MSDs^1,57,61,64,65^. Within this view, it might be relevant to draw attention to the MSDs in CeH. Prevalence of MSDs of the upper-cervical spine, which can be triggers for CeH, increases in office workers who are intensive computer users^66^. And, as a result of the global pandemic caused by COVID-19, prevalence of CeH is expected to increase due to immobilisation and digitalisation (e.g. home office work)^13^. Cervicogenic headache is presumed to be related to rather static, forced and/or prolonged habitual (cervical) postures, which can increase load on cervical musculoskeletal structures innervated by C1-C3 afferents^67–70^. Although cervical posture (e.g. FHP) has been extensively studied in CeH, little research was performed regarding SPV^11^. Based on the redundancy theory, the general lower SPV in the CeH-group compared to the control-group may imply a less balanced state. Finding an equilibrium in SPV might reflect an individual’s self-regulatory capacity to function in an optimal range. Such equilibrium corresponds to a state of homeostasis, which is critical to the sustainability of living organisms^71^. Since the key factor in sustainability is variability, variables related to such variability need to be identified^72^.

### Multiple biopsychosocial variables relate to spinal postural variability in patients with CeH

Previous studies already identified variables that influence intrinsic motor variability such as gender^72,73^, age^74^, experience (i.e. novice vs. experienced)^67^, fatigue^75^, musculoskeletal discomfort, and pain^64,76^. Several authors support the hypothesis that decreased motor variability is the motor response of patients suffering from long-term pain conditions (e.g. low back pain, unilateral patella-femoral pain, spastic hemiplegic cerebral palsy) in an attempt to limit movement as a protective mechanism^77–80^. However, we cannot confirm that headache-intensity was related to the lower SPV in the CeH-group. A first hypothesis for this finding could be the episodic character of CeH in our study, compared to chronic pain conditions in previous work^77–80^. Secondly, since cause-effect relations can be bidirectional, the lower SPV might have induced CeH.

The subjective individualized nature of experiencing pain, such as CeH, is created by a mix of factors unique to that person^81^. Both heterogeneity in clinical characteristics, and individual BPS characteristics such as gender, age, socio-economic status and psychosocial influences have been documented to influence the experience of pain, giving it a highly variable nature^77,78,82^. We extrapolated this knowledge to SPV, since we recently demonstrated that SPV also exists in patients with CeH^11^. The results of this current study revealed that SPV could be influenced by BPS variables in patients with CeH. Both intrinsic (stress, anxiety, sleep quality and pain processing) and extrinsic (desk-set-up, screen-time) variables seem to be significantly related to SPV.

Interestingly, several intrinsic and extrinsic BPS variables influenced SPV in the CeH-group, while LLx variability was the only outcome variable significantly different between groups. Two hypotheses might explain such difference in LLx variability. First, the lower LLx variability in the CeH-group (versus the control-group) might be the result of a protective strategy, and is consistent with the contemporary theory of motor adaptations to pain^3^. This theory suggests that pain induces postural adjustments to limit the amplitude of motion. Such adjustments can even occur at regions remote to the site of pain^3^. The results in our study could imply that participants with CeH are protecting their painful cervical spine by stiffening the lumbar region. Falla et al. (2017) already reported that participants with neck pain walked with a stiffer trunk^9^. Interestingly, in both studies a stiffening of the lumbar spine was seen^9^. This finding might indicate a bottom-up stiffening. However, future research is needed to analyse such hypothesis.

However, a second hypothesis should be considered. A minimum degree of variability is needed to create variation in joint load, muscle activity, and ligament stress as was postulated by the ‘variability-overuse hypothesis’^3,4,57,83^. The lower LLx variability in the CeH-group could place a cumulative load on the upper-cervical spine potentially activating a final common pathway (i.e. trigeminocervical nucleus caudatus) to induce CeH. More research is needed to further explore such finding, and its clinical relevance.

Time (the 30-min laptop-task) did not relate to SPV, implying that the course of SPV was rather flat and linear models (mean, SD) could be used^84^. Using non-linear models such as entropy analysis seems however needed to better understand the complexity of SPV^85,86^.

### Clinical relevance

Although this study could identify relations between BPS variables and SPV in patients with CeH, its clinical implication needs further exploration. To date, it is questioned whether SPV is causally related to CeH and if an optimal range of SPV exists^11,86,87^. Optimal variability seems, similar to homeostasis, a state in which a posture tends to remain balanced and stable. Therefore, determining the limits of SPV could provide clinical essential cut-offs for the transition of a healthy state, towards discomfort and eventually pain^76^.

### Limitations and recommendations

No Bonferroni corrections were applied because of the explorative nature. Therefore, and because of spectrum bias, results should be interpreted with caution.

Spinal postural variability can be assessed using several models. We used linear models (mean, SD) based on the characteristics of the data. It seems advised to additionally use non-linear models (e.g. entropy) to capture the complexity of SPV in future studies^85,86^. General residuals of the calibration method should have been provided to enable replications.

No cause-effect relationships between SPV and CeH can be extrapolated from the present analyses calling for longitudinal studies with adapted methodology, and possibly non-linear models.

Following these suggestions it should be questioned if a range of optimal variability exists, and if such range is specific to an individual^80^.

## Conclusion

Significant relations between SPV and BPS variables add new insights to the current knowledge on CeH. Spinal postural variability was lower, and related to more diverse BPS variables (i.e. pain processing, lifestyle, and psychosocial) in the CeH-group compared to the control-group. More research is needed into a possible causal relationship between SPV and CeH, and its clinical implication for management of this condition.

## Supplementary Information

Supplementary Informations.

## Abbreviations

BMI: Body Mass Index
BPS: BioPsychoSocial
CeH: Cervicogenic headache
CI: Confidence interval
CSI: Central sensitization inventory
DASS-21: Depression anxiety stress scale
ES: Effect size
FHP: Forward head posture
HIT-6: Headache impact test
ICC: Intraclass correlation coefficient
ICHD: International classification of headache disorders
IQR: Inter quartile range
L: Left
LCx: Lower-cervical spine
LLx: Lower-lumbar spine
LTx: Lower-thoracic spine
n: Number of participants
min: Minute
MSD(s): Musculoskeletal disorder(s)
N/A: Not applicable
NPRS: Numeric pain rating scale
PPT(s): Pressure pain threshold(s)
PSQI: Pittsburgh sleep quality index
R: Right
SD: Standard deviation
SPV: Spinal postural variability
UCx: Upper-cervical spine
ULx: Upper-lumbar spine
UTx: Upper-thoracic spine
VAS: Visual analogue scale
VIF: Variance inflation factor
