# Supplementary material for: Spinal postural variability relates to biopsychosocial variables in patients with cervicogenic headache
Source: Sci Rep. 2021 Jul 2;11:13783. doi: 10.1038/s41598-021-93138-3 (PMC8253805; doi:10.1038/s41598-021-93138-3)
Supplement: Supplementary file 1 — Supplementary Informations. [file 41598_2021_93138_MOESM1_ESM.docx]

**Spinal postural variability relates to biopsychosocial variables in patients with cervicogenic headache**

Sarah Mingels^1,2*^, Wim Dankaerts^2^, Ludo van Etten^3^, Liesbeth Bruckers^4^, and Marita Granitzer^1^

^1^ Hasselt University, REVAL Rehabilitation Research Centre, Biomedical Research Institute, Faculty of Rehabilitation Sciences, 3500, Hasselt, Belgium

^2^ Leuven University, Musculoskeletal Research Unit, Department of Rehabilitation Sciences, Faculty of Kinesiology and Rehabilitation Sciences, 3000, Leuven, Belgium

^3^ Zuyd Hogeschool, Department of Biometrics, 6419, Heerlen, The Netherlands

^4^ Hasselt University, Interuniversity Institute for Biostatistics and Statistical Bioinformatics, 3500, Hasselt, Belgium

* corresponding author: sarah.mingels@uhasselt.be

**Abbreviations**

**BMI:** Body Mass Index

**Ca:** Canthus

**CeH:** Cervicogenic Headache

**CFS:** Chronic Fatigue Syndrome

**CI:** Confidence Interval

**CVA**: Cerebro-Vascular Accident

**ES:** Effect Size

**FM:** Fibromyalgia

**ICHD:** International Classification Headache Disorders

**IPAQ:** International Physical Activity Questionnaire

**LOE:** Level of Education

**MS:** Multiple Sclerosis

**n:** number of participants

**NPRS:** Numeric Pain Rating Scale

**NSAID:** Non Steroid Anti Inflammatory

**OR:** Odds Ratio

**PT:** Physiotherapy

**SD:** Standard Deviation

**SPV:** Spinal Postural Variability

**TMD:** Temporo-mandibular disorder

**Tr:** Tragus

**V:** Vertical

**Supplementary file**

**Appendix A – Checklist participant recruitment**

The criteria used to in- and exclude participants are summarized in Table A.1.

**Table A.1**. Summary of inclusion- and exclusion criteria for the CeH-group and control-group.

| Inclusion CeH-group | Caucasian males, females between 18-55 years  BMI: 18.5 - 24.9  Dutch-speaking  Regular use of a laptop (minimum 7 hours/week) (based on the IPAQ)  Fulfilment of the diagnostic criteria for secondary CeH, ICHD-3 confirmed by a neurologist  Headache provocation during sitting activity |
| --- | --- |
| Inclusion control-group | Caucasian healthy* males, females between 18-55 years  BMI between 18.5 - 24.9  Dutch-speaking  Regular use of a laptop (minimum 7 hours/week) (based on the IPAQ) |
| Exclusion | Any other headache type or headache-related disorder (CeH-group)  Any headache type (Control-group)  Participation in another study (e.g. headache, postural education)  Pregnancy  Smoking  First headache onset > 50 years  Spinal surgery, trauma, headache therapy < 12 weeks prior to the study  Post-dural punction 2 weeks prior to the study  Cognitive limitations (Mini Mental State Examination test score < 30)  Serious pathology  - Musculoskeletal: hernia, disk prolapse, congenital spinal deviations  - Neurological: MS, Parkinson, epilepsy, CVA, myelopathy, myopathy, Benign Paroxysmal Position Vertigo, neurodegeneration, meningitis, encephalitis, dystonia  - Endocrine - System: FM, CFS, rheumatoid arthritis, infectious/inflammatory diseases  - Vascular: dysregulation blood pressure, postural hypotension, vertebra-basilar symptoms, …  Co-morbidities  - TMD, orthodontics, visual disorders, sleep apnoea, chronic sinusitis  - Psychiatric  - Hypermobility (Beighton ≥ 4)  - Medication-overuse: ergotamine, NSAID’s, opioids, acetylsalicylic acid, triptans, simple analgesics (> 10 days/month for > 3 months)  - Withdrawal of alcohol, caffeine or medication |

ICHD = International Classification Headache Disorders; BMI = Body Mass Index; NSAID = Non Steroid Anti Inflammatory; IPAQ = International Physical Activity Questionnaire; MS = Multiple Sclerosis; CVA = Cerebro-Vascular Accident; FM = Fibromyalgia; CFS = Chronic Fatigue Syndrome; TMD = Temporo-mandibular disorder; * = meaning no known painful conditions, serious pathologies (listed under exclusion).

**Appendix A – Flowchart of the recruitment and enrolment procedure**


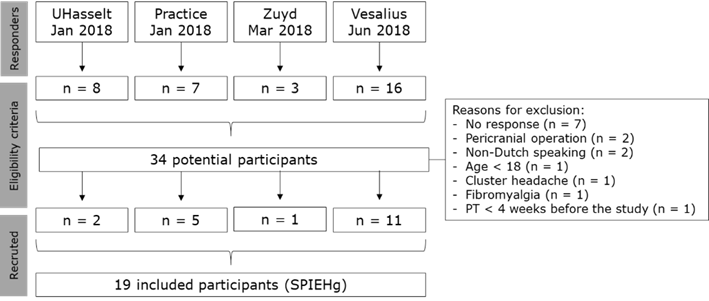


CeH-group

**Figure A.1.** Flowchart of recruitment of the CeH-group (n = number responders; PT = Physiotherapy).

***
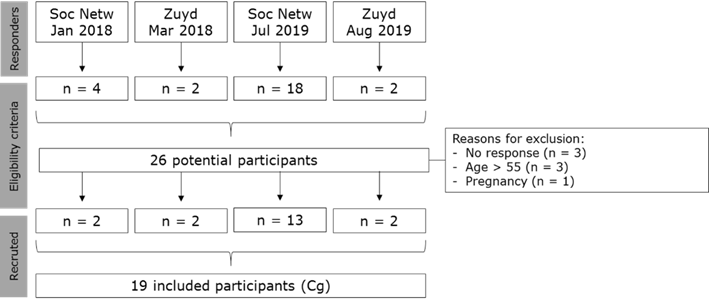
***

(Control-group)

**Figure A.2.** Flowchart of recruitment of the control-group (n = number responders; Soc Netw = Social Network).

**Appendix B – Interpretation secondary outcomes**

The ***Central Sensitization Inventory*** is scored based on the level of agreement on a 0-4 Likert scale (never, rarely, sometimes, often, always). Table B.1 provides a summary of the interpretation of the scores.

**Table B.1.** Interpretation and recommended cut-off scores for the Central Sensitization Inventory [32].

| Score | Interpretation |
| --- | --- |
| 0 to 29 | Subclinical symptoms of central sensitization |
| 30 to 39 | Mild symptoms of central sensitization |
| 40 to 49 | Moderate symptoms of central sensitization |
| 50 to 59 | Hard symptoms of central sensitization |
| 60 to 100 | Extreme symptoms of central sensitization |

The ***Numeric Pain Rating Scale*** consists of an 11-point scale, ranging from 0 (no pain) to 10 (worst pain imaginable [34]. The minimal detectable change amounts 2.1, the meaningful clinically important change 2.5 [35-37]. Table B.2 provides a summary of the interpretation of the scores.

**Table B.2.** Interpretation and recommended cut-off scores for the Numeric Pain Rating Scale [34].

| Score | Interpretation |
| --- | --- |
| ≤ 3 | Mild pain |
| 4 to 6 | Moderate pain |
| ≥ 7 | Severe pain |

The ***Pittsburgh Sleep Quality Index*** is scored 0 (no problem) to 3 (serious problem). A total score exceeding 5/21 on the Pittsburgh Sleep Quality Index indicates poor sleep quality. Table B.3 provides a summary of the interpretation of the scores.

**Table B.3.** Interpretation and recommended cut-off scores for the lifestyle characteristics.

| Lifestyle characteristics | Interpretation | | |
| --- | --- | --- | --- |
| Pittsburgh Sleep Quality Index [38]  ≤ 5  6-7  ≥ 8 | Optimal  Borderline  Poor | |  |
| Physical activity time/week  ≤ 2 hours  1 to 2 times, ≥ 30 minutes  minimal 3 times, ≥ 30 minutes | Low  Moderate  High | |  |
| Screen-time hours/week  < 7 hours  7 to 14 hours  14 to 21 hours  > 21 hours | Little  Substantiate  Moderate  Severe |  |  |
| Sedentary-time hours/day  0 hours  < 3 hours  3 to 6 hours  ≥ 7 hours | No  Little  Moderate  Severe |  |  |

Items on the ***Depression Anxiety Stress Scale-21*** are scored according a Likert-scale (0 = did not apply to me at all, and 3 = applied to me very much or most of the time). Table B.4 provides a summary of the interpretation of the scores on each subscale.

**Table B.4.** Interpretation and recommended cut-off scores for the subscales of the Depression Anxiety Stress Scale-42 [44].

|  | Depression | Anxiety | Stress |
| --- | --- | --- | --- |
| Normal | 0-9 | 0-7 | 0-14 |
| Mild | 10-13 | 8-9 | 15-18 |
| Moderate | 14-20 | 10-14 | 19-25 |
| Severe | 21-27 | 15-19 | 26-33 |
| Extremely severe | > 28 | > 20 | > 34 |

Scores the Depression Anxiety Stress Scale-21 need to be multiplied by two.

Questions on the ***Headache Impact Test-6*** are completed based on the level of agreement on a 0-4 Likert scale (never, rarely, sometimes, very often, always), and numerically converted: 6, 8, 10, 11, and 13, respectively. The impact of headache on daily life depends on the total score which varies between 36 and 78. Table B.5 provides a summary of the interpretation of the scores.

**Table B.5.** Interpretation and recommended cut-off scores for the Headache Impact Test-6 [46,48].

| Score | Interpretation |
| --- | --- |
| ≤ 49 | No to little impact of headache on daily life |
| 50-55 | Headache seems to affect daily life |
| 56-59 | Headache has a significant impact on daily life |
| ≥ 60 | Headache has a very heavy impact on daily life |

|  |
| --- |

|  |
| --- |

**Appendix C – Interpretation primary outcomes**

The paragraphs below provide detailed information on the methodology concerning the spinal measurements and were extracted from our previous work [11].

***Data collection, processing and analysis***

The principal researcher was responsible for the data collection. Motion analysis was performed via 12 infrared Bonita T10 and two video cameras at a sample rate of 100Hz (low-pass Woltring filter). The biomechanical model, a custom labelling skeleton template, was created with Vicon Nexus and evaluated (2016-2017, Zuyd Hogeschool). This model was derived from numerous studies that examined human sitting posture. Seven reflective markers were placed on the following anatomical landmarks to model the spine: left tragus and canthus, C7, T6, T12, L3 and S2 spinous processes. Data processing was conducted in Vicon Nexus (software, Nexus 2.1.1). Processed trials were converted to pseudonymized c3d-files, exported to a custom developed data analysis programme (MATLAB version R2019b, Natick, Massachusetts: The MathWorks Inc.), and converted to angles by an independent researcher. Spinal posture was recorded each minute for five seconds during the 30-minute-laptop-task. Angles were calculated based on the mean of the middle three seconds of the five second recording. Left sagittal angles were calculated for the: the upper- and lower-cervical (UCx, LCx), thoracic (UTx, LTx), and lumbar (ULx, LLx) spine (Table C.1, Figure C.1). Spinal postural variability (SPV) was deducted from these measurements.

**Table C.1.** Overview of the determination of spinal angles of the biomechanical model.

| Angle | Markers (section) | Angle (°) |
| --- | --- | --- |
| Upper-cervical (α) | Tragus, Canthus (8 mm) | Between line through markers on tragus – canthus and vertical |
| Lower-cervical (β) | Tragus (8 mm), C7 (14 mm) | Between line through markers on C7 – tragus and vertical |
| Upper-thoracic (γ) | C7, T6 (14 mm) | Between line through markers on T6 – C7 and vertical |
| Lower-thoracic (δ) | T6, T12 (14 mm) | Between line through markers on T12 – T6 and vertical |
| Upper-Lumbar (ε) | T12, L3 (14 mm) | Between line through markers on L3 - T12 and vertical |
| Lower-lumbar (ζ) | L3, S2 (14 mm) | Between line through markers on S2 - L3 and vertical |

° = degrees; mm = millimetre.

Spinal angles were calculated based on following method, an example is given for angle $\alpha$(UCx):

Let Canthus (Ca) be $\left( \begin{matrix} x_{\mathrm{ca}} \\ y_{\mathrm{ca}} \\ z_{\mathrm{ca}} \end{matrix} \right)$, Tragus (Tr) $\left( \begin{matrix} x_{\mathrm{tr}} \\ y_{\mathrm{tr}} \\ z_{\mathrm{tr}} \end{matrix} \right)$ and Vertical (V) $\left( \begin{matrix} 0 \\ 0 \\ 1 \end{matrix} \right)$, then vector TrCa is $\left( \begin{matrix} x_{\mathrm{tr}}-x_{\mathrm{ca}} \\ y_{\mathrm{tr}}-y_{\mathrm{ca}} \\ z_{\mathrm{tr}}-z_{\mathrm{ca}} \end{matrix} \right)$. Now $\alpha$ can be calculated using equation the equation : $\cos\left( \alpha\right)=\frac{TrCa \cdot V}{\left\| TrCa \right\| \left\| V \right\|}$, with $\left\| a \right\|=\sqrt{x_{a}^{2}+y_{a}^{2}+z_{a}^{2}}$.

**
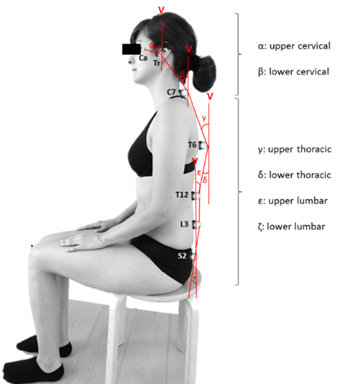
**

**Figure C.1.** Biomechanical model with marker placement and angle (α to ζ) determination (V = Vertical; Ca = Canthus; Tr = Tragus). System accuracy < 1 mm (Zuyd Hogeschool, 2016).

***Procedure***

*Marker placement and static calibration*. Concerning the CeH-group, a condition to be measured was a score of < 3 on the 11-point numeric pain rating scale (NPRS) for headache-intensity on the test day. Participants were asked not to take analgesics, muscle relaxants, and caffeine-containing beverages 24 hours prior to the measurements. Prophylactic treatment(s) remained unchanged. Measurements were performed in a real-life set-up with a constant room temperature of 25° Celsius at the motion laboratory of Zuyd Hogeschool (Heerlen, The Netherlands). A static calibration of the sitting posture was performed for each participant before testing started. During the calibration participants were seated on a desk chair without back support, both feet on the floor, upper legs horizontal, lower legs vertical, feet parallel and shoulder width apart on the ground, arms uncrossed on thighs (Figure C.1). After degreasing the skin, anatomical landmarks were located by manual palpation, and marked by an experienced (> 10 years) manual therapist (= principal researcher). The most prominent bony anatomical landmarks were selected to limit soft tissue artefacts. Next, reflective markers were fixed on the skin using double-sided adhesive tape at the previously described anatomical landmarks. Individual spinal postures were expressed relative to their static sitting posture. The principal researcher performed the test procedure for both the CeH-group and control-group.

*Workstation setup.* The capture volume of the Vicon system was 15 m². A standard desk (height 74 cm; depth 80 cm; width 120 cm) (Bureau voor Normalisatie NBN-EN527) with laptop (HP® ProBook 650 or HP® 6470b Elitebook) and a height-adjustable office chair without back rest were placed in the centre of the capture volume. The position of the laptop, inclination of the screen and position of the chair referred to the ground could be individually adjusted. The position of the setup had to ensure visibility of each marker by at least two infrared cameras.

*Test procedure.* Participants were asked to *‘sit as you normally do’*. No further instructions, nor feedback was given. Hereafter a 35-minute customized laptop-task was performed. Posture was not recorded throughout the first five minutes to familiarize the participant with the work station. During the 30-minute-laptop-task the habitual spinal posture was recorded for five seconds every minute, the first measurement was defined as ‘t0’. SPV was deducted from these measurements.

For the customized laptop-task the most common on- and offline laptop activities were selected (browsing, editing and typing). Participants completed nine standardised questions (Microsoft® Word 2016), four questions accentuated typing words and numbers through open questions, five questions required an internet-search (Google Chrome™ or Mozilla Firefox®), the use of a computer-mouse was not allowed. The test procedure was executed and guided by the principal researcher.

**Appendix D – Details mixed models**

Table D.1 provides a summary of the outcomes of the mixed model for repeated measures.

**Table D.1.** Summary of the outcomes of the mixed model for repeated measures.

| UCx CeH-group Control-group p ^Δ^ | | | |
| --- | --- | --- | --- |
| Baseline habitual UCx (°), (SD) [CI]  Covariance parameters, estimate (SD) [CI]  Intercept  Time  Variance  Structure (AR)1 | 77.9 (15.6) [69.6;86.2]  10.83 (8.43) [3.61;124.91]  0.05 (0.03) [0.02;0.26]  51.93 (4.16) [44.65;61.16]  0.33 (0.05) [0.24;0.43] | 82.4 (14.3) [75.;89.8]  73.01 (35.58) [33.85;259.69]  0.08 (0.05) [0.03;0.49]  125.58 (8.44) [110.56:143.91]  0.12 (0.05) [0.02;0.21] | .4^‡^ |
| Fixed effects, estimate (SD)  Intercept  Baseline  Time  Group  Group x time (Cg) | 29.46 (10.23)  0.67 (0.12)  -0.08 (0.08)  -1.12 (2.7)  0.01 (0.12) | | .**008**  **< .0001**  .3  .68  .91 |
| LCx CeH-group Control-group p ^Δ^ | | | |
| Baseline habitual LCx (°), (SD) [CI]  Covariance parameters, estimate (SD) [CI]  Intercept  Time  Variance  Structure (AR)1 | 63.9 (16) [56;71.9]  29.84 (14.02) [14.15;98.88]  0.04 (0.03) [0.01;0.26]  45.31 (3.56) [39.07;53.17]  0.32 (0.05) [0.22;0.42] | 66.5 (12.3) [60.1;72.8]  61.16 (31.9) [27.16;242]  0.02 (0.04) [0.001;1.1]  193.11 (13.25) [169.55;221.97]  0.24 (0.04) [0.15;0.33] | .6^†^ |
| Fixed effects, estimate (SD)  Intercept  Baseline  Time  Group  Group x time (Cg) | 32.24 (55.96)  0.55 (0.09)  0.07 (0.07)  -2.88 (2.92)  0.56 (0.09) | | **< .0001**  **< .0001**  .27  .33  .69 |
| UTx CeH-group Control-group p ^Δ^ | | | |
| Baseline habitual UTx (°), (SD) [CI]  Covariance parameters, estimate (SD) [CI]  Intercept  Time  Variance  Structure (AR)1 | 33.8 (10.7) [28.3;39.3]  11.88 (5.36) [21.66;101.35]  0.03 (0.02) [0.02;0.10]  14.23 (1.05) [12.39;16.53]  0.25 (0.05) [0.15;0.35] | 37.8 (8.7) [33.3;42.2]  40.52 (15.39) [5.76;37.14]  0.04 (0.02) [0.02;0.11]  21.56 (1.46) [18.97;24.75]  0.18 (0.05) [0.08;0.27] | .25^†^ |
| Fixed effects, estimate (SD)  Intercept  Baseline  Time  Group  Group x time (Cg) | 11.48 (3.27)  0.73 (0.1)  0.03 (0.05)  -0.2 (1.97)  -0.008 (0.08) | | **.003**  **< .0001**  .56  .92  .91 |

| LTx CeH-group Control-group p ^Δ^ | | | |
| --- | --- | --- | --- |
| Baseline habitual LTx (°), (SD) [CI]  Covariance parameters, estimate (SD) [CI]  Intercept  Time  Variance  Structure (AR)1 | 9.6 (5.8) [6.8;12.5]  1.73 (1.67) [0.49;54.42]  0.03 (0.01) [0.01;0.08]  12.16 (1) [10.41;14.38]  -0.04 (0.05) [0.29;0.48] | 8.8 (7.4) [4.9;12.8]  10.06 (4.42) [4.96;30.24]  0.01 (0.007) [0.004;0.07]  27.03 (1.71) [23.96;30.71]  0.39 (0.05) [-0.13;0.06] | .72^†^ |
| Fixed effects, estimate (SD)  Intercept  Baseline  Time  Group  Group x time (Cg) | 2.76 (0.88)  0.91 (0.08)  0.005 (0.05)  -0.03 (0.06)  0.01 (0.02) | | **.004**  **< .0001**  .91  .57  .51 |
| ULx CeH-group Control-group p ^Δ^ | | | |
| Baseline habitual ULx (°), (SD) [CI]  Covariance parameters, estimate (SD) [CI]  Intercept  Time  Variance  Structure (AR)1 | 4.1 (8.5) [-0.3;8.5]  3.25 (1.92) [1.33;16.62]  0.01 (0.006) [0.004;0.05]  10.89 (0.8) [9.47;12.65]  0.3 (0.04) [0.21;0.4] | 7.6 (12.4) [1;14.2]  4.17 (3) [1.48;36.36]  0.01 (0.008) [14.24;19.56]  16.58 (1.34) [14.24;19.56]  0.39 (0.05) [0.3;0.48] | .56^‡^ |
| Fixed effects, estimate (SD)  Intercept  Baseline  Time  Group  Group x time (Cg) | 0.15 (0.62)  0.77 (0.06)  0.03 (0.05)  1.18 (0.92)  -0.006 (0.05) | | .81  **< .0001**  .46  .21  .9 |
| LLx CeH-group Control-group p ^Δ^ | | | |
| Baseline habitual LLx (°), (SD) [CI]  Covariance parameters, estimate (SD) [CI]  Intercept  Time  Variance  Structure (AR)1 | 11.7 (11.6) [5.5;17.9]  56.02 (21.43) [29.83;141.27]  0  84 (9.16) [72.42;98.62]  1 | 13.9 (13.3) [6.5;21.2]  150.18 (59.55) [78.47;394.88]  0.24 (0.12) [0.11;0.91]  130.77 (11.44) [110.36;157.44]  0.98 | .62^†^ |
| Fixed effects, estimate (SD)  Intercept  Baseline  Time  Group  Group x time (Cg) | 17.11 (3.84)  -0.04 (0.01)  0.03 (0.07)  -4.14 (4.99)  -0.07 (0.16) | | **< .0001**  **.0007**  .62  .41  .35 |

SD = Standard Deviation; CI = 95% Confidence Interval; ‡ = Mann-Whitney test; † = unpaired t-test; Δ = p-value deducted from the mixed model with random and fixed effects; Bold numbers = p < .05.

Table D.2 provides a summary of the influence of time on variance (SPV), deducted from the mixed model).

**Table D.2.** Summary of the influence of time (independent variable) on variance in the CeH-group and control-group, deducted from the mixed model.

| Posture | CeH-group  Variance (SD) [CI]  Time included | CeH-group  Variance (SD) [CI]  Time excluded | Control-group  Variance (SD) [CI]  Time included | Control-group  Variance (SD) [CI]  Time excluded |
| --- | --- | --- | --- | --- |
| UCx | 52.11 (4.21) [44.74;61.46] | 51.97 (4.17) [44.68;61.21] | 125.58 (8.44) [110.56:143.91] | 125.51 (8.82) [110.51;143.8] |
| LCx | 45.13 (3.52) [38.95;52.91] | 45.26 (3.54) [39.04;53.09] | 193.11 (13.25) [169.55;221.97] | 192.8 (13.2) [169.34;221.53] |
| UTx | 14.26 (1.05) [12.4;16.56] | 14.23 (1.05) [12.39;16.53] | 21.56 (1.46) [18.97;24.75] | 21.57 (1.46) [18.97;24.75] |
| LTx | 10.55 (1.86) [7.68;15.41] | 10.34 (1.75) [7.62;14.84] | 27.03 (1.71) [23.96;30.71] | 15.14 (5.5) [8.28;36.08] |
| ULx | 10.81 (0.79) [9.42;12.54] | 10.89 (0.8) [9.47;12.65] | 16.58 (1.34) [14.24;19.56] | 16.55 (1.33) [14.22;19.51] |
| LLx | 83.84 (6.77) [72;98.88] | 83.75 (6.55) [72.27;98.22] | 130.77 (11.44) [110.36;157.44] | 141.17 (12.83) [119.04;170.12] |

SD = Standard Deviation; CI = 95% Confidence Interval.

**Appendix E – Relations between the independent variables and the outcomes**

Relations between age, BMI, their interaction (age x BMI), level of education, employment, headache-intensity and spinal postural variability were analyzed through multiple linear regression (continuous outcome). An overview of the statistical significance (p-values) of these relations is provided in Tables D.1 and D.2

**Table E.1.** Summary of relations between independent variables and outcomes in the CeH-group (n = 18).

| Outcome | Age (p) | BMI (p) | Age x BMI (p) | LOE (p) | Job (p) | Headache-intensity (p) |
| --- | --- | --- | --- | --- | --- | --- |
| SPINAL POSTURAL VARIABILITY | | | | | |  |
| SD UCx | .81 | .11 | .79 | .72 | .92 | .63 |
| SD LCx | .46 | .45 | .6 | .09 | .47 | .55 |
| SD UTx | .87 | .76 | .62 | .91 | .71 | .87 |
| SD LTx | .62 | .34 | .82 | .47 | .45 | .89 |
| SD ULx | .65 | .9 | .83 | .77 | .66 | .61 |
| SD LLx | .67 | .34 | .17 | .77 | .44 | .85 |

p-values were deducted from multiple regression models; SD = Standard Deviation; LOE = Level of Education; Headache-intensity = NPRS post-laptop-task.

**Table E.2.** Summary of relations between independent variables and outcomes in the control-group (n = 18).

| Outcome | Age (p) | BMI (p) | Age x BMI (p) | LOE (p) | Job (p) |
| --- | --- | --- | --- | --- | --- |
| SPINAL POSTURAL VARIABILITY | | | | | |
| SD UCx | .62 | .22 | .05 | .62 | .16 |
| SD LCx | .11 | .72 | .05 | .33 | .08 |
| SD UTx | .85 | .85 | .83 | .99 | .99 |
| SD LTx | .05 | .06 | .36 | .69 | .05 |
| SD ULx | .05 | .55 | .55 | .95 | .1 |
| SD LLx | .46 | .28 | .05 | .21 | .62 |

p-values were deducted from multiple regression models; SD = Standard Deviation; LOE = Level of Education.

**Appendix F – Pain processing, psychosocial, and lifestyle characteristics**

**Table F.1.** Summary: Pain processing characteristics in the CeH-group (n = 18) and control-group (n = 18).

| Central Sensitization Inventory | | | |
| --- | --- | --- | --- |
| Dimensions, n (%) | **CeH-group** | **Control-group** | **p^‡^ (OR)** |
| Subclinical (0 - 29)  Mild (30 - 39)  Moderate (40 - 49)  Hard (50 - 59)  Extreme (60 - 100) | 4 (22.2)  5 (27.8)  7 (38.9)  2 (11.1)  0 | 10 (55.6)  3 (16.7)  5 (27.8)  0  0 | .18 |
| Subclinical vs. mild, moderate, hard extreme combined | 4 vs. 14 | 10 vs. 8 | **.04 (4.4)** |

| Pressure Pain Threshold (kPa/cm² (SD) [CI]) | | | |
| --- | --- | --- | --- |
| Muscle | **CeH-group** | **Control-group** | **p^†^** (ES) |
| Suboccipital Left | 206.5 (78.8) [166.2;246.8] | 332.6 (268.2) [199.3;466] | **.03** (0.64) |
| Suboccipital Right | 179.3 (75.1) [140.8;217.7] | 273.3 (203.2) [172.3;374.4] | **.009** (0.61) |
| Erector spine Left | 428.1 (236.2) [310.6;545.5] | 584 (229.6) [469.9;698.2] | **.04** (0.67) |
| Erector spine Right | 418 (185) [323.3;512.7] | 596.2 (214.3) [489.6;702.8] | **.008** (0.89) |
| Tibialis anterior Left | 365.3 (122.9) [302.3;425.2] | 613.2 (263.1) [482.4;744] | **.001** (1.21) |
| Tibialis anterior Right | 397.3 (144.2) [323.5;471.1] | 626.9 (284.4) [485.5;768.4] | **.007** (1.03) |

SD = Standard Deviation; CI = 95% Confidence Interval; n = number participants; ‡ = contingency table for categorical variables (Fisher’s exact test); ^†^ = unpaired t-test; Bold numbers = p < .05; ES = Effect Size; OR = Odds Ratio.

**Table F.2.** Summary: Psychosocial and lifestyle characteristics in the CeH-group (n = 18) and control-group (n = 18).

| Psychosocial characteristics | CeH-group | Control-group | p^‡^ |
| --- | --- | --- | --- |
| Depression, Anxiety, Stress Scale-21, n (%)  Depression  Normal (0 – 9)  Mild (10 – 13)  Moderate (14 – 20)  Severe (21 - 27)  Extreme (≥ 28)  Anxiety  Normal (0 – 7)  Mild (8 – 9)  Moderate (10 – 14)  Severe (15 – 19)  Extreme (≥ 20)  Stress  Normal (0 – 14)  Mild (15 – 18)  Moderate (19 – 25)  Severe (26 – 33)  Extreme (≥ 34) | 10 (55.6)  2 (11.1)  2 (11.1)  3 (16.7)  1 (5.6)  7 (38.9)  6 (33.3)  2 (11.1)  1 (5.6)  2 (11.1)  7 (38.9)  4 (22.2)  1 (5.6)  3 (16.7)  3 (16.7) | 14 (77.8)  0  3 (16.7)  1 (5.6)  0  9 (50)  5 (27.8)  3 (16.7)  1 (5.6)  0  7 (38.9)  3 (16.7)  8 (44.4)  0  0 | .33  .82  .**01** |
| Headache Impact Test-6, n (%)  Little/none (≤ 49)  Substantiate (50 - 55)  Moderate (56 - 59)  Severe (> 60)  p^1^ within the group | 2 (11.1)  1 (5.6)  6 (33.3)  9 (50)  **.008** | 18 (100)  0  0  0  **< .0001** | **< .0001** |

| Lifestyle characteristics | CeH-group | Control-group | p^‡^ (ES) |
| --- | --- | --- | --- |
| Pittsburgh Sleep Quality Index, n (%)  Optimal (≤ 5)  Borderline (6 – 7)  Poor (≥ 8) | 7 (38.9)  5 (27.8)  6 (33.3) | 11 (61.1)  3 (16.7)  4 (22.2) | .5 |
| Sleep quality (10 cm VAS), mean (SD)  [CI] | 6.6 (1.2)  [5.9;7.2] | 2.6 (2.2)  [1.5;3.7] | **< .0001^*^** (0.95) |
| Sleep duration (hours/night), mean (SD)  [CI] | 6.9 (0.9)  [6.5;7.4] | 7.4 (1.4)  [6.7;8.1] | .25* |
| Physical activity hours/week, n (%)  Low (≤ 2h)  Moderate (1 to 2 times, ≥ 30 min)  High (minimal 3 times, ≥ 30 min) | 7 (38.8)  9 (50)  2 (11.1) | 13 (72.2)  2 (11.1)  3 (16.7) | .05 |
| Screen-time hours/week, n (%)  Little (< 7h)  Substantiate (7 – 14h)  Moderate (14 – 21h)  Severe (> 21h) | 1 (5.6)  5 (27.8)  1 (5.6)  11 (61.1) | 1 (5.6)  6 (33.3)  2 (11.1)  9 (50) | .89 |
| Sedentary-time: free-time hours/day, n (%)  No (0 h)  Little (< 3 h)  Moderate (3 – 6 h)  Severe (≥ 7 h) | 1 (5.6)  7 (38.9)  9 (50)  1 (5.6) | 0  4 (22.2)  13 (72.2)  1 (5.6) | .63 |
| Sedentary-time: work hours/day, n (%)  No (0 h)  Little (< 3 h)  Moderate (3 – 6 h)  Severe (≥ 7 h) | 1 (5.6)  3 (16.7)  7 (38.9)  7 (38.9) | 1 (5.6)  1 (5.6)  10 (55.6)  6 (33.3) | .62 |

SD = Standard Deviation; CI = 95% Confidence Interval; h = hours; n = number participants; VAS = 10 cm Visual Analogue Scale (0 = best, 10 = worst sleep quality); ‡ = contingency table for categorical variables (Fisher’s exact test); * = Mann Whitney Test; Bold numbers = p < .05; ES = Effect Size.
